# Potential and application of *Fusobacterium nucleatum* in the diagnosis and treatment of colorectal cancer

**DOI:** 10.3389/fmicb.2025.1652702

**Published:** 2025-09-30

**Authors:** Xin He, Qing Zhao, Jianhong Zhang, Jing Shi, Ningyi Wan, Bin Tang, Bo Tian, Pu Li

**Affiliations:** ^1^Department of Clinical Laboratory, Chongqing University Jiangjin Hospital, Chongqing, China; ^2^Department of Clinical Laboratory, The Second Affiliated Hospital of Chongqing Medical University, Chongqing, China; ^3^Department of Clinical Laboratory, The First Affiliated Hospital of Chongqing Medical University, Chongqing, China; ^4^Chongqing Mental Health Center, Chongqing, China

**Keywords:** *F. nucleatum*, colorectal cancer, therapeutic monitoring, prognostic evaluation, biomarker

## Abstract

Colorectal cancer (CRC), as a globally prevalent malignant tumor, relies on in-depth analysis of tumor microenvironment regulation mechanisms for precision diagnosis and treatment. *Fusobacterium nucleatum* (*F. nucleatum*), a key carcinogenic bacterium, has been revealed in recent studies to play multidimensional roles in CRC initiation, progression, and metastasis. This review systematically summarizes the progress of Fn applications in CRC full-cycle management: (1) In the diagnostic field, Fn detection technology based on fecal samples has developed into a new non-invasive screening strategy. Cohort studies show its diagnostic performance (AUC 0.82–0.89), with significant correlations to tumor stage (III/IV stage OR = 2.87), lymph node metastasis (HR = 1.94), and reduced 5-year survival rate (35% vs. 62%); (2) For therapeutic monitoring, dynamic Fn load changes can predict chemotherapy (OR = 0.63) and immunotherapy responses (PFS extended by 2.1 months); (3) In prognostic evaluation, metagenomic analysis shows that high Fn abundance is closely related to TNM staging (C-index 0.81 vs. 0.69) and recurrence risk (AUC = 0.88). Notably, a nomogram model integrating Fn biomarkers can improve the predictive accuracy of the traditional TNM staging system by 17.3%. Although existing evidence supports the clinical translational value of Fn, its standardized detection protocols, threshold setting, and targeted intervention strategies (such as antibiotic therapy and phage therapy) still require validation through multi-center prospective studies. This review provides evidence-based medical evidence for the application of Fn in CRC precision medicine by integrating multi-omics data.

## 1 Introduction

### 1.1 Epidemiology of colorectal cancer and disease background related to *F. nucleatum*

**FIGURE 1:**
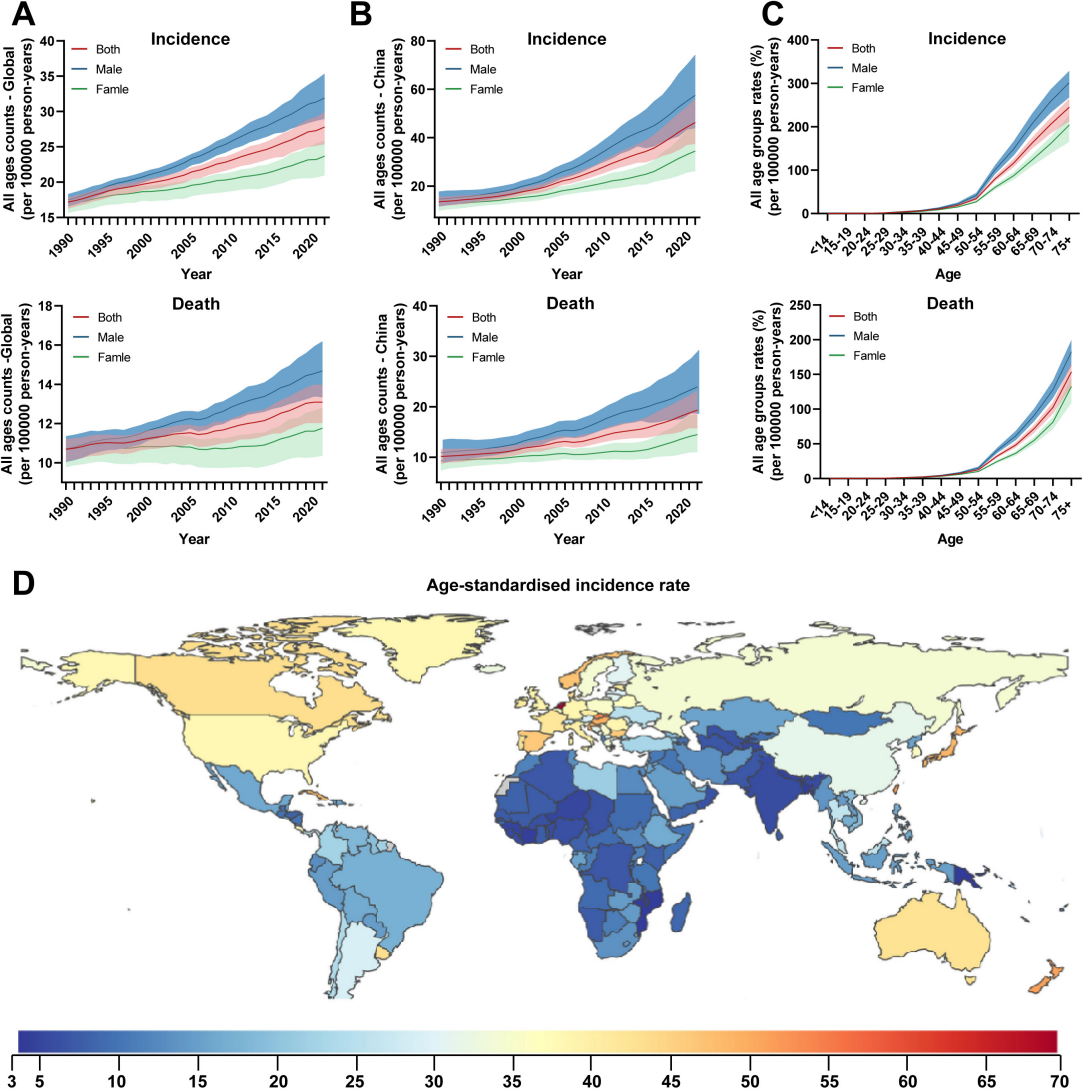
Global temporal patterns of colorectal cancer burden, 1990–2021. **(A)** All-age counts. The global annual number of newly diagnosed CRC cases exceeds one million and shows an upward trend; **(B)** Age-standardized rates. In China, the number of newly diagnosed cases reached 517,100 in 2022; **(C)** All age groups rates in 2021. **(D)** Geographical distribution of age-standardized rates of colorectal cancer in 2021. Data source: Global Burden of Diseases, Injuries, and Risk Factors Study 2024 (Institute for Health Metrics and Evaluation, 2024).

Colorectal cancer (CRC) is the third most common malignant tumor worldwide, with over one million newly diagnosed cases annually and a rising trend (Figure 1A). Its incidence is significantly higher in China, Europe, and North America compared to the global average (Figure 1D). Disease risk increases sharply with age, with a significant rise in incidence after 50–55 years and mortality after 45–50 years (Figure 1C). Due to improved living standards and lifestyle changes in China, CRC incidence and mortality continue to increase (Figure 1B). According to 2022 Chinese cancer statistics, CRC ranks second in incidence and fourth in mortality among malignant tumors, with approximately 517,100 new cases and 240,000 deaths annually (Han et al., 2024). *F. nucleatum* is a core microorganism in oral dental plaque (Bolstad et al., 1996; Borisy and Valm, 2021), widely distributed in the human digestive system, reproductive system, and other sites. It is associated with various diseases, including periodontitis, pancreatitis, CRC, pelvic inflammatory disease, and adverse pregnancy outcomes (Han, 2015; Swidsinski et al., 2011; Xu and Han, 2022). Multiple studies indicate significantly elevated *F. nucleatum* abundance in CRC tissue and fecal samples, suggesting its close association with CRC development (Castellarin et al., 2012; Wong and Yu, 2019). The detection rate of this bacterium in CRC tissues is much higher than in normal tissues, indicating its potential as a diagnostic biomarker or therapeutic target.

### 1.2 Core role and clinical significance of *F. nucleatum* in colorectal cancer

As a Gram-negative anaerobic bacterium, *F. nucleatum* plays a critical role in CRC initiation, recurrence, metastasis, and drug resistance. It participates in CRC progression through mechanisms such as activating inflammatory responses, promoting tumor cell proliferation and invasion, and inducing resistance to chemotherapy and immunotherapy. Systematic studies on *F. nucleatum* abundance in intestinal tissues and related molecular expressions are valuable for elucidating CRC pathogenesis, optimizing diagnostic and therapeutic strategies, and assessing prognosis. Existing reviews often focus on basic research and lack clinical guidance. This study integrates the association, pathogenic mechanisms, and clinical applications of *F. nucleatum* and CRC, constructing a review framework with both theoretical depth and clinical practicality to inform CRC precision medicine. The study emphasizes the following directions: (1) exploring interactions between *F. nucleatum*, intestinal microbiota, and host factors; (2) developing targeted therapeutic strategies against *F. nucleatum*; (3) promoting standardization of detection methods and normalization of data analysis to ensure scientific rigor and reproducibility. These efforts will provide new insights for personalized CRC treatment and ultimately improve patient survival and prognosis.

### 1.3 Literature search strategy

To systematically review research progress on *F. nucleatum* in colorectal cancer, this study employed a structured literature search approach. Databases searched included PubMed, Embase, Web of Science, and China National Knowledge Infrastructure (CNKI), with a search period from 1 January 2010, to 30 June 2024. A combined keyword strategy was used: (“*Fusobacterium nucleatum*” OR “*F. nucleatum*”) AND (“colorectal cancer” OR “CRC”) AND (“diagnosis” OR “therapy” OR “prognosis”). Inclusion criteria were as follows: (1) original research papers; (2) provision of explicit detection methods or clinical data; (3) sample size ≥ 50 cases. Exclusion criteria were as follows: (1) reviews, conference abstracts, or case reports; (2) non-human studies; (3) duplicate published data. Finally, the included literature underwent independent screening and cross-validation by two researchers.

## 2 Biological characteristics and functions of *F. nucleatum* in the gut

### 2.1 Classification and phylogeny of *F. nucleatum*

*F. nucleatum* belongs to the family Fusobacteriaceae. This bacterium is anaerobic but can still grow in environments with oxygen levels up to 6% (Bolstad et al., 1996). Early observations in the human oral cavity identified fusiform microorganisms, and the Fusobacterium genus was isolated based on sensitivity to dyes and antibiotics (Baird-Parker, 1957). Active strains that ferment amino acids, produce acetate and butyrate, and exhibit limited sugar-degrading activity are classified as *F. nucleatum* (Yu et al., 2022). Subsequent studies on 16S genomics have suggested that the common ancestor of Fusobacterium was Leptotrichia, which underwent adaptive radiation during evolution, diverging into three main lineages and five major clades (Manson McGuire et al., 2014). Based on 16S rRNA gene sequence analysis, it can be further classified into four subspecies: *nucleatum*, *animalis*, *vincentii* (including fusiforme), and *polymorphum* (Nie et al., 2015). These subspecies have been found in clinical tissues and fecal samples of patients with CRC, with a significant increase in *F. nucleatum* (Bi et al., 2022; Table 1).

**TABLE 1:** Fusobacterial strain-level associations with colorectal cancer.

Strain name	Subspecies	Primary association	References
*Fusobacterium nucleatum*	*Nucleatum*	As an early diagnostic marker for CRC	Liu et al., 2021
		Promotes the occurrence of CRC	Rubinstein et al., 2019
		Induces metastasis of CRC	Zhang et al., 2022
*Fusobacterium animalis*	*Animalis*	Mediates immune regulation in CRC	Lamprinaki et al., 2021
		Induces inflammatory responses and promotes the progression of CRC	Ye et al., 2017
		Associated with higher colorectal cancer-specific mortality rates and specific somatic mutation genes	Borozan et al., 2022
*Fusobacterium vincentii*	*Vincentii*	Can be isolated from CRC tissues and saliva	Castellarin et al., 2012; Komiya et al., 2019; Manson McGuire et al., 2014
*Fusobacterium polymorphum*	*Polymorphum*	Detectable in CRC saliva samples	Morsi et al., 2022

### 2.2 Ecological niche and symbiotic relationships of *F. nucleatum*

In the intestinal tract, *F. nucleatum* forms complex interactions with host microbiota, influencing its colonization and pathogenicity, thereby affecting gastrointestinal immunity and metabolism. When microbial diversity is high and butyrate-producing bacteria (e.g., Faecalibacterium, Roseburia) are abundant, *F. nucleatum* struggles to obtain sufficient nutrients and adhesion sites. However, microbiota dysbiosis caused by antibiotics, high-fat diets, or inflammatory bowel disease (IBD) weakens the “colonization resistance” of commensal bacteria, providing a window for *F. nucleatum* colonization (Dewan et al., 2025). Evidence from the oral-intestinal axis indicates that *F. nucleatum* can form “corncob-like” co-aggregates with oral resident bacteria Streptococcus sanguinis, utilizing its filamentous structure to more easily penetrate the mucus layer and colonize colorectal mucosa (Sun et al., 2019). *F. nucleatum* can exploit ornithine (ArcD-dependent) excreted by Streptococcus gordonii as a nitrogen source, accelerating its own proliferation (Sun et al., 2019). *F. nucleatum* acts as a bridge between early and late colonizers in dental plaque by forming biofilms (Borisy and Valm, 2021). Signaling molecules from *P. gingivalis* can accelerate *F. nucleatum* biofilm formation (Yamaguchi-Kuroda et al., 2023). In biofilm form, *F. nucleatum* exhibits enhanced virulence and invasiveness, enabling it to invade multi-layered epithelial collagen matrices and survive under aerobic conditions (Gursoy et al., 2010), thereby disrupting gastrointestinal immune and metabolic homeostasis.

### 2.3 Pathogenic mechanisms of *F. nucleatum* in colorectal cancer

**FIGURE 2:**
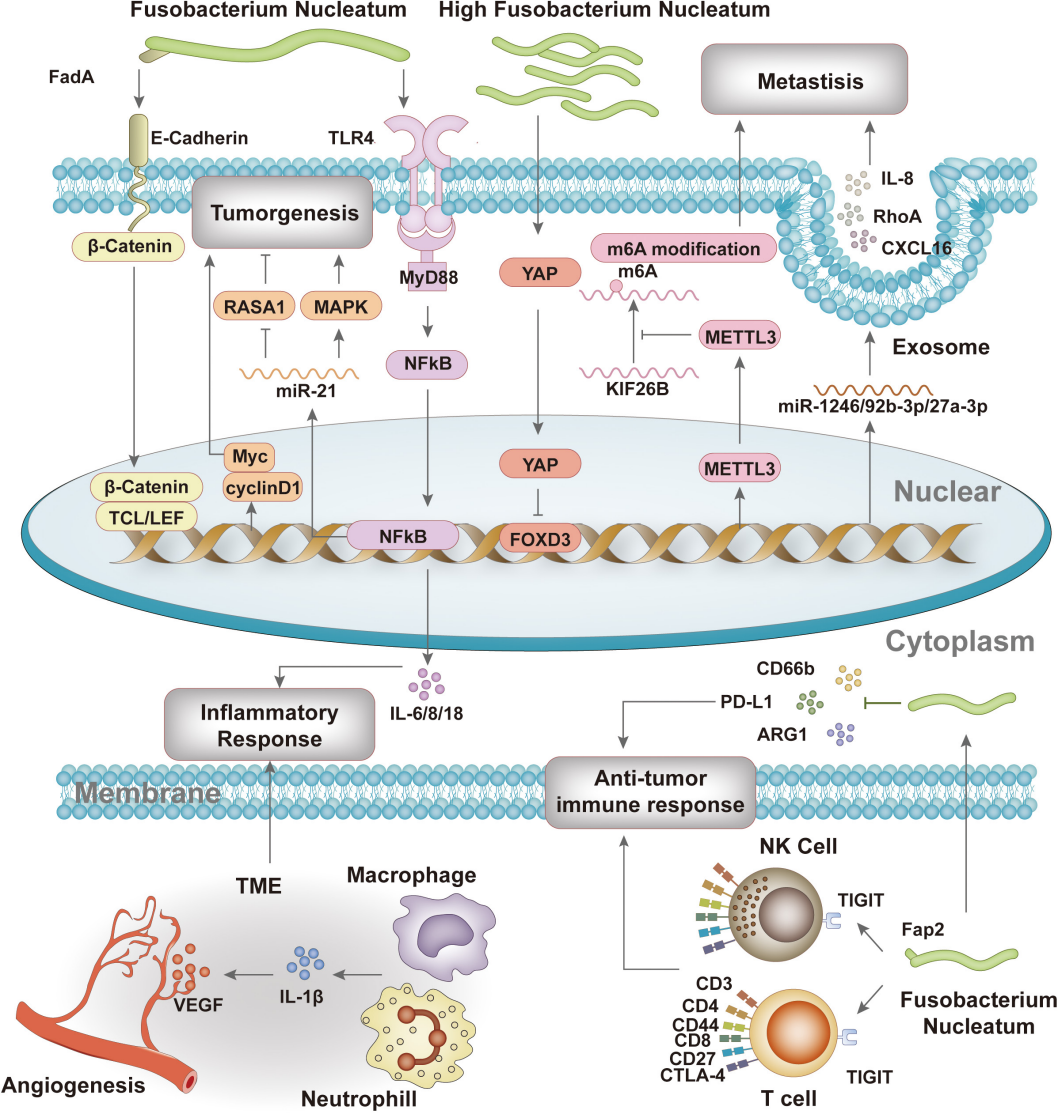
The pathogenic mechanisms of *F. nucleatum* in colorectal cancer.

Studies have confirmed that *F. nucleatum* participates in the formation, progression, and treatment response of colorectal cancer through a series of complex mechanisms (Figure 2).

Firstly, *F. nucleatum* binds to colorectal epithelial cells via adhesin FadA and E-cadherin, promoting tumor cell proliferation and invasion (Manson McGuire et al., 2014; Rubinstein et al., 2013). Gal-Gal-NAc overexpression in colorectal cancer enables *F. nucleatum* recognition and binding, leading to its accumulation in tumor tissues (Abed et al., 2016). *F. nucleatum* abundance changes are significant in colorectal cancer patients, highlighting its potential as a biomarker for screening and diagnosis (Kwong et al., 2018; Liang et al., 2020; Lin et al., 2022; Yu J. et al., 2017). It also reduces m^6^A modification in CRC cells, enhancing invasiveness (Chen et al., 2022), shifts central carbon metabolism in tumor cells, and promotes CRC cell invasion (Ternes et al., 2022). *F. nucleatum* activates TLR4 signaling, leading to NF-κB activation and increased miR-21 expression, which promotes tumor metastasis (Yang et al., 2017).

Secondly, *F. nucleatum* activates inflammatory responses and immune evasion, inhibiting host immune responses and promoting tumor development. It induces pro-inflammatory factors such as NF-κB, IL-6, and IL-8 (Queen et al., 2021; Rubinstein et al., 2013), increases inflammation-related gene expression (Galeano Niño et al., 2022), and exists in immunosuppressive microecological niches, reducing CD4 and CD8 levels while upregulating CD66b+, ARG1, and CTLA4 (Galeano Niño et al., 2022). *F. nucleatum*’s Fap2 protein interacts with the inhibitory receptor TIGIT on NK and T cells (Gur et al., 2015), and upregulates PD-L1 expression in CRC cell lines, promoting immune evasion (Galeano Niño et al., 2022; Gao et al., 2023).

Additionally, *F. nucleatum* influences the tumor microenvironment by regulating angiogenesis and metastasis. Inflammatory responses induce IL-1β production, which activates endothelial cells to produce pro-angiogenic factors, promoting angiogenesis and tumor progression (Jagielska et al., 2012; Nakao et al., 2005). *F. nucleatum* alters miRNA and chemokine expression in host cells, delivered via exosomes, increasing cell migration and tumor metastasis (Guo et al., 2020). It also upregulates KRT7-AS, regulating CRC cell lymph node migration (Chen et al., 2020, 2022). Regarding treatment, *F. nucleatum* levels correlate with colorectal cancer treatment response. Increased *F. nucleatum* levels are associated with improved response to PD-L1 blockade therapy, possibly by activating STING signaling and increasing PD-L1 expression (Gao et al., 2021). However, *F. nucleatum* may also impair CD8^+^ T cell immunity, reducing sensitivity to anti-PD-1 mAb and increasing immunotherapy resistance (Jiang et al., 2023). It initiates protective autophagy via the TLR4 pathway, enhancing chemotherapy resistance (Zheng et al., 2019).

*F. nucleatum* promotes CRC progression through dual mechanisms: (1) *F. nucleatum* mediates signaling pathways, including the FadA/E-cadherin/β-catenin regulatory axis and METTL3/m^6^A modification, to regulate CRC proliferation and invasion capabilities, thereby directly influencing CRC progression; (2) *F. nucleatum* induces the production of pro-inflammatory factors such as NF-κB, IL-6, and IL-8 while modulating PD-L1 expression to affect inflammatory and anti-tumor immune responses, thereby indirectly regulating CRC progression.

### 2.4 Genetic research progress and tool development of *F. nucleatum*

Recent advances in genetic technologies have significantly deepened the understanding of *F. nucleatum*-CRC association mechanisms. At the genomic level, whole-genome sequencing and functional annotation revealed key differences among subspecies: for example, the *F. animalis* genome harbors a unique fnp gene cluster closely associated with its tumor metastasis-promoting capacity, while the *nucleatum* subspecies (*F. nucleatum*) carries more adhesin genes related to oral colonization. Comparative genomic analysis further identified differentially expressed genes among subspecies (e.g., fadA adhesin, gal-galNAc receptor) that directly influence their pathogenic potential in the intestine (Ma et al., 2023; Queen et al., 2021). At the transcriptomic level, RNA sequencing has constructed high-resolution global RNA profiles of Fn subspecies during early, mid-exponential, and early stationary growth phases, aiding in elucidating functional characteristics at different disease progression stages (Ponath et al., 2021).

In multi-omics integration, combining transcriptomics and metabolomics revealed that Fn promotes cell proliferation by modulating amino acid biosynthesis, central carbon metabolism, protein digestion/absorption, and other metabolic pathways in CRC cells (Wu et al., 2023). At the genetic engineering level, tools including CRISPR interference (CRISPRi) systems, suicide plasmid-based gene inactivation systems, replicative plasmid-based gene expression control systems, and transposon-based random mutagenesis systems have become critical strategies for studying Fn pathogenicity. These tools enabled targeted silencing of key virulence genes (e.g., fadA, gal-galNAc), facilitating validation of their roles in tumor cell adhesion and invasion (Guan et al., 2025; Zhou et al., 2024). The establishment of these genetic tools not only clarified subspecies-specific pathogenic mechanisms (e.g., *F. animalis* exhibits significantly stronger metastasis-promoting capacity than *F. nucleatum*) but also advanced the development of precision intervention strategies against *F. nucleatum*, such as subspecies-specific antigen-based vaccine design and inhibitors targeting key metabolic pathways. Integrating multi-omics technologies with organoid models holds promise for further dissecting the dynamic microbiota-host interaction network, providing novel targets for CRC precision medicine.

## 3 Application of *F. nucleatum* in the diagnosis of colorectal cancer

### 3.1 Quantitative and localization analysis of *F. nucleatum*

**FIGURE 3:**
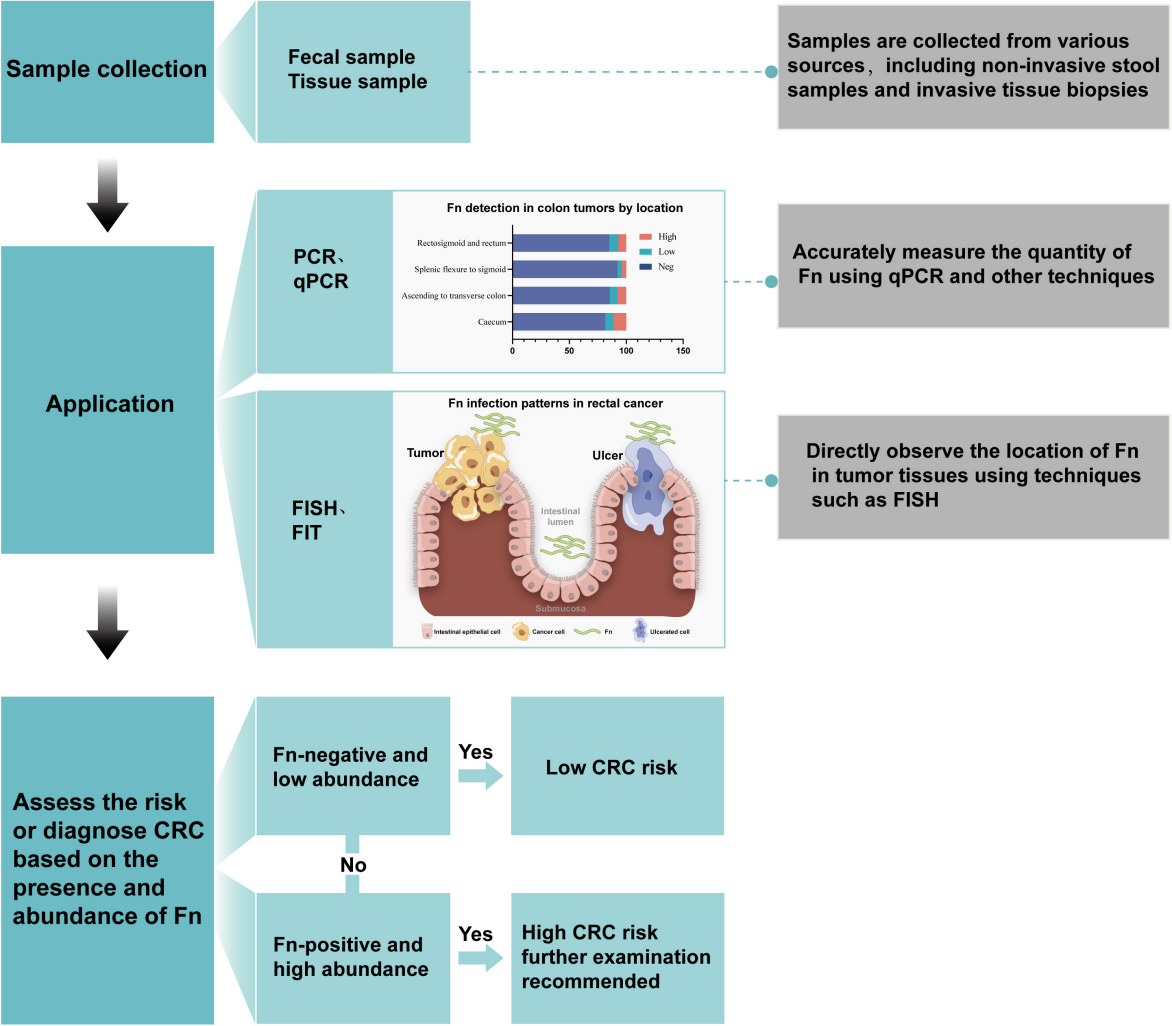
Application process of *F. nucleatum* in the diagnosis of colorectal cancer.

Quantitative and localization analysis of *F. nucleatum* is a significant direction in current colorectal cancer research. It aims to deepen the understanding of its role in the occurrence and development of colorectal cancer and may also provide new strategies for future diagnosis and treatment. Currently, commonly used methods for detecting *F. nucleatum* include culture, PCR, qPCR, FISH, FIT, etc. PCR can determine the presence and abundance of *F. nucleatum* by detecting its specific genes or 16S rRNA sequences. The diagnostic workflow consists of three stages: (1) sample preprocessing; (2) quantitative PCR (qPCR) using FadA gene-specific primers; and (3) bioinformatics analysis. The application process is shown in Figure 3.

#### 3.1.1 Culture

Isolation and identification of *F. nucleatum* from clinical samples is a reliable diagnostic method that provides insight into the pathogen. However, routine detection through culture is challenging due to difficulties in sample transportation and culturing, as well as the low abundance of *F. nucleatum* in the intestinal tract and interference from other bacterial flora. We developed an IMB assay for direct isolation and culture of *F. nucleatum* from human feces, with a sensitivity of 10^3^ CFU mL^–1^, but it is challenging and suitable only for experienced microbiologists. To address this, we further developed a selective chromogenic solid medium that promotes *F. nucleatum* growth while inhibiting other bacteria and facilitates identification through color differences. This method improves the positive rate of isolation and culture in clinical specimens.

#### 3.1.2 Serological test

A serological test can detect *F. nucleatum*-specific antibodies in serum, saliva, and urine. It is inexpensive, non-invasive, and convenient to detect IgG antibodies using laboratory-based serology, and is especially suitable for large-scale epidemic research. The presence of specific antibodies in the blood can persist for several weeks following *F. nucleatum* infection. Hence, a positive serum test for antibodies cannot serve as the basis for an ongoing infection. In conclusion, serology is not recommended as a routine method for diagnosing *F. nucleatum* infection, but it can be helpful when combined with other methods.

#### 3.1.3 Fecal immunochemical test

The fecal immunochemical test (FIT) is non-invasive, rapid, and convenient for sampling. The stability of the fecal bacterial composition can last up to 144 h, with low levels of bacterial contamination. Furthermore, bacterial biomarkers can be stably detected in FIT, making it suitable for CRC screening (Grobbee et al., 2020). FIT performs well in detecting colonic lesions in symptomatic patients but has limited overall diagnostic efficacy (Liang et al., 2021). Combining FIT with qPCR or sDNA for the detection of other biomarkers can significantly improve the sensitivity of *F. nucleatum* detection (Liang et al., 2021; Wong et al., 2017).

#### 3.1.4 Molecular methods

Quantitative analysis, primarily using qPCR and high-throughput sequencing, measures the abundance of *F. nucleatum* in CRC tissues or fecal samples. These techniques have revealed that *F. nucleatum* levels are significantly higher in CRC patients compared to healthy individuals and correlate with tumor malignancy, staging, and prognosis (Guo et al., 2020; Yang et al., 2017). Quantitative analysis can also predict treatment response in CRC patients (Lee et al., 2021).

Detection rates of *F. nucleatum* in CRC tissues vary across studies (Table 2). The variability in detection rates of colonic adenomas and colorectal cancers may stem from differences in microbiota colonization sites and primer specificity. During colorectal cancer progression, *F. nucleatum* exhibits heterogeneous distribution, with significantly higher abundance in superficial regions compared to deep regions, leading to differences in detection rates between colonic adenomas and CRC tissues (Yamamoto et al., 2021). Additionally, primers targeting the FadA adhesin gene (e.g., those used in Yamamoto et al., 2021) demonstrate 2.3-fold higher sensitivity than universal 16S rRNA primers, explaining discrepancies among studies (OR = 3.82, 95% CI 1.25–11.7). qPCR can also detect *F. nucleatum* in fecal samples, enabling non-invasive detection (Tunsjø et al., 2019). Fecal metagenomic analysis has identified gene markers for CRC, including two validated by qPCR in an independent CRC patient cohort, highlighting the potential for early-stage CRC diagnosis (Yu J. et al., 2017). In a study on cancer-related fecal microbial markers, *F. nucleatum* showed a specificity of 76.9%, a sensitivity of 69.2%, and an ROC of 0.737 for predicting CRC (Eklöf et al., 2017).

**TABLE 2:** Detection rates of *F. nucleatum* in CRC tumor tissues across different studies.

Total cases	Positive cases	Positive percentage	Detection method	Detection samples	References
1,069	134	13%	PCR	Carcinoma tissue	Mima et al., 2016
812	99	12%	PCR	Carcinoma tissue	Ugai et al., 2023
724	99	14%	qPCR	Carcinoma tissue	Haruki et al., 2020
116	54	47%	qPCR	Adenocarcinoma tissue	Lo et al., 2022
254	143	56%	qPCR	Adenocarcinoma tissue	Serna et al., 2020

The variation in detection rates may stem from: (1) differences in primer design (e.g., 16S rRNA gene V3–V4 region vs. FadA gene-specific primers) (Mima et al., 2016; Serna et al., 2020); (2) heterogeneous distribution (adenoma tissue vs. cancerous tissue) (Yamamoto et al., 2021).

#### 3.1.5 Fluorescence *in situ* hybridization

Localization analysis uses immunohistochemistry and FISH to determine the location of *F. nucleatum* in CRC tissues. FISH can detect *F. nucleatum*, visualize its interaction with tumor cells, and show if bacteria are adhered to or have invaded cells (Guo et al., 2021; Li et al., 2016). Studies found that *F. nucleatum* closely interacts with tumor cells and may invade them. Galeano Niño et al. (2022) used RNAscope-FISH to show *F. nucleatum* within CRC epithelial cells, associated with higher immune cell presence in *F. nucleatum*-positive samples. *F. nucleatum* is mainly in the tumor region, with clear contact with tumor cells, potentially affecting CRC cell transcription and gene expression, promoting proliferation and invasion.

However, quantitative and localization analysis results may be affected by sample collection, processing, and detection technique sensitivity/specificity. Researchers must control experimental conditions for accuracy and reliability. Technological advancements are yielding new methods, which will further reveal *F. nucleatum*’s role in CRC and provide new diagnostic and treatment strategies.

### 3.2 Correlation and sensitivity of *F. nucleatum* with colorectal cancer

The high enrichment of *F. nucleatum* in colorectal cancer tissues suggests its role in cancer development. Its abundance correlates with malignancy grade, clinical stage, and prognosis, with high levels indicating poorer prognosis and higher recurrence risk. *F. nucleatum* enhances cancer cell stemness, invasion, and metastasis, promoting tumor progression. It also affects the tumor microenvironment and regulates immune cell function and distribution. While the correlation is significant, the specific mechanism is not fully understood (see Table 3 for more information). Future research should explore *F. nucleatum*’s role in cancer development, its interactions with the tumor microenvironment and immune system, and develop targeted therapeutic strategies. A deeper understanding will provide new insights and methods for colorectal cancer diagnosis and treatment.

**TABLE 3:** Relationship between *F. nucleatum* and clinical characteristics of colorectal cancer.

Clinical feature	Population	Methodology	Sample Size	Sample resource	Results	References
Tumor Size	China	Droplet digital PCR	100	CRC patient tumor tissue	Tumor size is significantly larger in the high *F. nucleatum* group compared to the low *F. nucleatum* group (*P* = 0.004).	Yamaoka et al., 2018
	China	qPCR	92	CRC patient tumor tissue	The quantity of *F. nucleatum* is positively correlated with the American Joint Committee on Cancer (AJCC) staging and tumor size.	Yu T. et al., 2017
	Japan	qPCR	200	CRC patient tumor tissue	Tumor size in the *F. nucleatum*-positive group (median 30 mm; range 4–100 mm) is significantly larger than in the *F. nucleatum*-negative group (median 8 mm; range 2–82 mm) (*P* < 0.001).	Yamamoto et al., 2021
Lymph Node Metastasis	Canada	qPCR	97	CRC patient tumor tissue	CRC tissues with higher *F. nucleatum* abundance are more likely to develop lymph node metastasis (*P* = 0.0035).	Castellarin et al., 2012
	China	qRT-PCR	79	CRC patient fecal, HCT-166 Cell, LoVo Cell	The abundance of *F. nucleatum* is significantly increased in CRC patients with lymph node metastasis. *F. nucleatum* infection promotes lymph node metastasis and *in vitro* metastasis of CRC.	Chen et al., 2020
Metastasis	China	qPCR	77	CRC patient tumor tissue	In metastatic CRC patients, the abundance of *F. nucleatum* in stage IV shows an increasing trend compared to stage I. *F. nucleatum* infection significantly enhances the migration and invasion capabilities of CRC cells.	Xu et al., 2021
	China	Transwell	–	HCT116 Cell	*F. nucleatum* increases the invasiveness and metastatic capability of CRC cells.	Chen et al., 2022; Guo et al., 2020; Kong et al., 2021, 2023
Tumor Invasion Depth	Japan	qPCR	53	CRC patient tumor tissue	The expression rates of *F. nucleatum* on the tumor surface and in the tumor depth are 45.7% and 32.6%, respectively.	Yamamoto et al., 2021
	United states, Germany	qPCR	22	CRC patient fecal	Patients with high *F. nucleatum* abundance are three times more likely to be diagnosed with rectal cancer compared to colon cancer (OR = 3.01; 95% CI, 1.06–8.57). Patients with high fecal *F. nucleatum* abundance have a fivefold higher risk of being diagnosed with rectal cancer compared to right-sided colon cancer (OR = 5.32; 95% CI, 1.23–22.98).	Eisele et al., 2021
TNM staging	China	qPCR	78	CRC patient tumor tissue	*F. nucleatum* infection is significantly associated with advanced TNM staging.	Kong et al., 2021
	Germany	qPCR	105	CRC patient tumor tissue	*F. nucleatum* infection is not statistically significantly associated with TNM staging.	Eisele et al., 2021
	USA, Canada, Australia, New Zealand, Austria	qPCR	1,994	CRC patient tumor tissue	Tumors diagnosed at stage II (OR = 1.77) or stage III (OR = 1.84) are more likely to be positive for *F. nucleatum* compared to stage I tumors.	Borozan et al., 2022
	Japan	qPCR	200	CRC patient tumor tissue	Detection rates are associated with pathological staging: 5.9% in adenomas (7/118), 26.1% in stage 0 (6/23), 35.1% in stage I/II (13/37), and 81.8% in stage III/IV (8/22).	Yamamoto et al., 2021
	United States	qPCR	1096	CRC patient tumor tissue	The amount of *F. nucleatum* DNA in CRC tissues is significantly associated with tumor invasion depth, AJCC staging, and tumor differentiation (*p* < 0.05).	Mima et al., 2016
	China	qPCR	116	CRC patient tumor tissue	Compared to *F. nucleatum*-negative CRC patients, those with *F. nucleatum* infection have higher odds for TNM staging (OR = 2.19, CI 1.03–4.64), lymph node involvement (OR = 2.19, CI 1.03–4.64), and distant metastasis (OR = 24.47, CI 0.89–22.51).	Lo et al., 2022
	China	Droplet digital PCR	100	CRC patient tumor tissue	The copy number of *F. nucleatum* is significantly higher in stage IV patients compared to those in stages I–III.	Yamaoka et al., 2018

OR, odds ratio; HR, hazard ratio.

## 4 Application of *F. nucleatum* in monitoring the therapeutic effect of CRC

### 4.1 Association between *F. nucleatum* and chemotherapy and immunotherapy

*F. nucleatum* plays a pivotal role in CRC chemotherapy. Studies indicate that *F. nucleatum* significantly promotes the development of chemotherapy resistance in colorectal cancer. *F. nucleatum* facilitates CRC resistance by activating autophagy and inhibiting pyroptosis and ferroptosis (Li et al., 2024; Wang et al., 2024; Yu T. et al., 2017). Additionally, *F. nucleatum* promotes the secretion of hsa_circ_0004085 via exosomes, influencing endoplasmic reticulum stress and thereby enhancing chemotherapy resistance in CRC (Hui et al., 2024).

With the widespread application of immunotherapy in colorectal cancer, the association between *F. nucleatum* burden and treatment efficacy has become a research focus. Recent studies reveal a bidirectional regulatory relationship between *F. nucleatum* load and response to immune checkpoint inhibitor (ICI) therapy: on one hand, it upregulates PD-L1 expression through m^6^A modification of IFIT1 or activation of the STING pathway, while recruiting IFN-γ^+^CD8^+^ tumor-infiltrating lymphocytes (TILs), thereby enhancing tumor sensitivity to PD-L1 therapy (OR = 3.82, 95% CI 1.25–11.7) (Gao et al., 2021, 2023); on the other hand, succinate produced by the bacterium reduces levels of IFN-γ, TNF-α, and chemokines such as CCL5/CXCL10 in the tumor microenvironment, inhibiting CD8^+^ T cell infiltration and leading to resistance against anti-PD-1 monoclonal antibodies (HR = 2.14, 95% CI 1.07–4.28) (Jiang et al., 2023). This contradictory phenomenon may relate to differences in *F. nucleatum* colonization sites, which drive activation of distinct intracellular and extracellular signaling pathways in CRC, resulting in opposing immunotherapeutic regulatory effects.

Given the critical role of *F. nucleatum* in CRC chemotherapy and immunotherapy resistance, studies have begun exploring *F. nucleatum*-targeted therapeutic strategies to enhance treatment sensitivity. Research indicates that antibiotic treatment with metronidazole can reduce intestinal *F. nucleatum* and restore immunotherapy sensitivity (Jiang et al., 2023; Wang et al., 2023). Oral or intravenous administration of azide-modified phage covalently linked to dextran nanoparticles, which inhibit *F. nucleatum* growth, significantly improves the efficacy of first-line CRC chemotherapy (Zheng et al., 2019). The use of tubercidin I (TBI) simultaneously enhances dendritic cell (DC) vaccine efficacy and suppresses *F. nucleatum* infection, thereby improving immunotherapy outcomes (Tong et al., 2023). The development of *F. nucleatum*-targeted therapeutic approaches holds promise for reducing CRC resistance.

### 4.2 *F. nucleatum* as an indicator for assessing treatment effectiveness

Beyond its critical role in CRC drug resistance, *F. nucleatum* also demonstrates clinical potential in monitoring treatment efficacy, post-therapy recurrence rates, and mortality. *F. nucleatum* is significantly associated with poor response to chemotherapy/immunotherapy, increased post-treatment recurrence, and elevated patient mortality in CRC (Jiang et al., 2023; Wang et al., 2024; Yu T. et al., 2017). Studies indicate that *F. nucleatum* significantly elevates chemotherapy-specific mortality in colon cancer patients [hazard ratio (HR) = 1.92, 95% confidence interval (CI): 1.07–3.45] (Borozan et al., 2022); *F. nucleatum* positivity markedly increases recurrence risk in chemotherapy-treated CRC patients (HR = 7.5, 95% CI: 3.0–19.0; *P* < 0.001) (Serna et al., 2020).

## 5 Multidimensional application of *F. nucleatum* in CRC prognostic assessment

**FIGURE 4:**
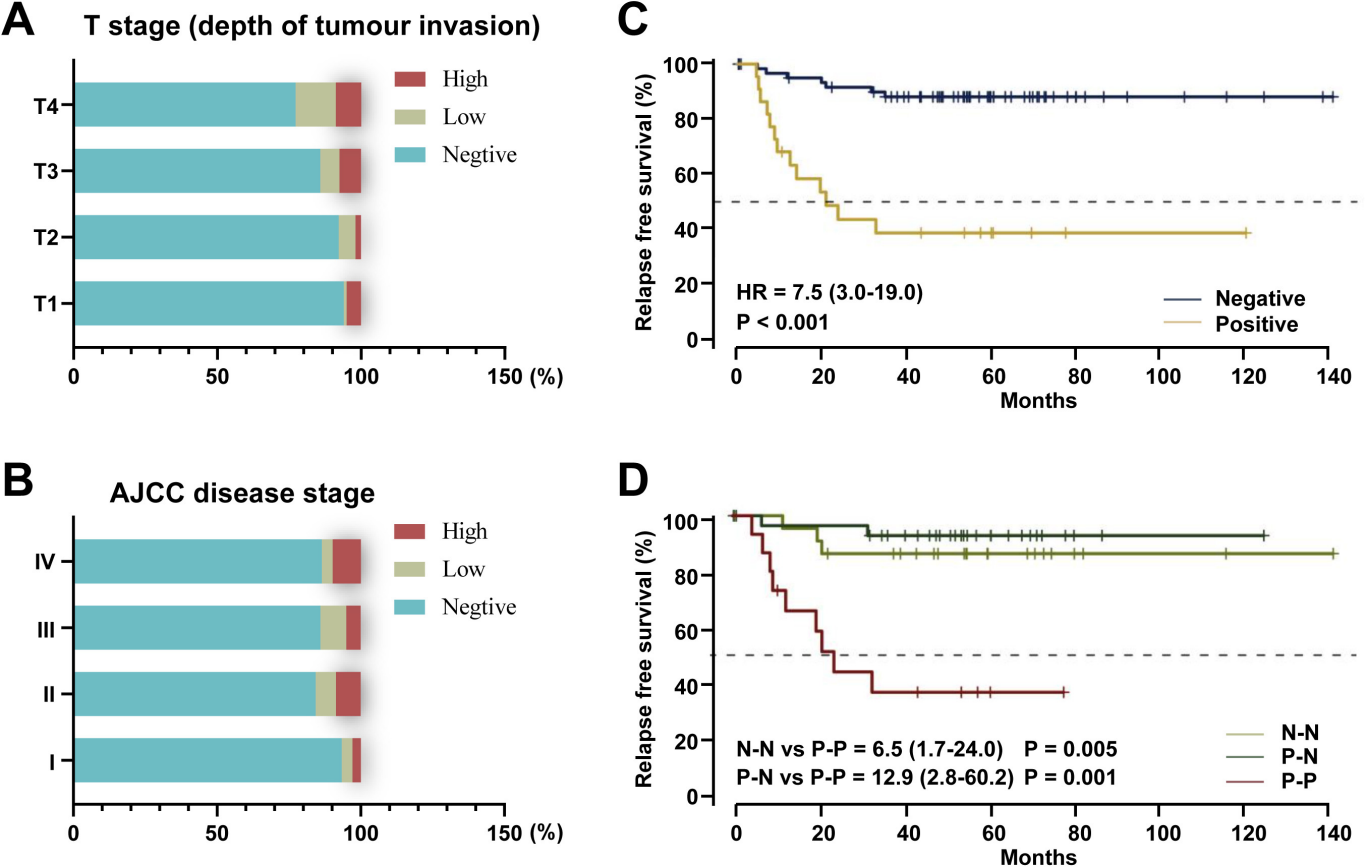
The role of *F. nucleatum* in the prognostic evaluation of colorectal cancer. **(A)** *F. nucleatum* detection by tumor infiltration depth (T1, submucosa; pT2, muscularis propria; T3, subserosa; T4, serosa or other organs). **(B)** *F. nucleatum* detection by AJCC disease stage (Mima et al., 2016). **(C)** Treated cohort by *F. nucleatum* status in post-nCRT tumor samples. **(D)** Paired treated cohort grouped according to the shift in *F. nucleatum* status between pre-nCRT and post-nCRT paired samples. N-N: patients who maintained negative *F. nucleatum* status before and after treatment. P-N: patients in whom *F. nucleatum* was negative after treatment. P-P: patients with a positive *F. nucleatum* status in both samples (Serna et al., 2020). HR: hazard ratio.

*F. nucleatum* is closely associated with prognostic evaluation in colorectal cancer (CRC). Research shows that *F. nucleatum* abundance increases progressively with tumor invasion depth (T1–T4) (*P* < 0.001) (Figure 4A); its levels correlate positively with AJCC staging (C-index = 0.81) (Figure 4B); among patients receiving neoadjuvant chemotherapy, those with high *F. nucleatum* levels exhibit the lowest relapse-free survival (RFS) (Figures 4C–D). Meta-analysis of multi-center data from China, the US, and Germany revealed that, except for Germany, all cohorts showed significant association between high *F. nucleatum* abundance and shortened overall survival (OS) (Table 4). The inconsistency in Germany may relate to cohort heterogeneity in demographic-molecular backgrounds and “negative confounding” from treatment modal differences across countries (Kunzmann et al., 2019; Lee et al., 2019). Multivariate analysis demonstrated a gradient elevation in CRC-specific mortality risk for *F. nucleatum*-low (HR = 1.25, 95% CI: 0.82–1.92) and *F. nucleatum*-high (HR = 1.58, 95% CI: 1.04–2.39) patients compared to negatives (Mima et al., 2016). Notably, *F. nucleatum* exhibits higher multivariate HR in left-sided colon cancer, suggesting anatomic site-specific prognostic value (Mouradov et al., 2023). Given its prognostic significance, researchers are exploring the integration of *F. nucleatum* into prognostic models. A proposed model combining *F. nucleatum* with four other bacterial species outperforms traditional markers like CEA and lymph node metastasis (baseline C-index = 0.69; C-index + M5 = 0.78) (Huh et al., 2022).

**TABLE 4:** Relationship between *F. nucleatum* and clinical prognosis of colorectal cancer.

Population	Methodology	Sample size	Sample resource	Results	References
United States	PCR	106	CR patient tumor tissue	Colorectal cancer-specific mortality: *F. nucleatum* low-positive vs. *F. nucleatum* negative: HR 1.25 (CI 0.82–1.92, *P* < 0.05) Colorectal cancer-specific mortality: *F. nucleatum* high-positive vs. *F. nucleatum* negative: HR 1.85 (CI 1.04–2.39, *P* < 0.05) *F. nucleatum* is significantly associated with high MSI: OR 5.22, CI 2.86–9.55, *P* < 0.05	Mima et al., 2016
Japan	qPCR	125	CRC patient tumor tissue	Patients with higher levels of *F. nucleatum* DNA and miR21 have a greater risk of poor prognosis.	Yang et al., 2017
South Korea	RT-PCR	99	CRC patient tumor tissue	In stage III CRC, *F. nucleatum*-positive patients have lower disease-free survival and overall survival (OS) compared to *F. nucleatum*-negative patients (DFS *P* = 0.0019, OS *P* = 0.0304).	Kim et al., 2023
China	qRT-PCR	98	CRC patient tumor and adjacent non-tumor tissues	*F. nucleatum* is significantly associated with shorter OS time in CRC patients.	Zhang et al., 2022
China	*In vivo* optical imaging	14	Mouse rectal fecal samples	The liver metastasis rates in PBS-treated mice and *F. nucleatum*-treated mice are 26.67% and 66.67%, respectively.	Yin et al., 2022
China	qRT-PCR	258	CRC patient tumor and adjacent non-tumor tissues	*F. nucleatum* is significantly associated with shorter survival time in CRC.	Chen et al., 2022
China	qPCR	228	CRC patient tumor tissue	*F. nucleatum* is associated with poorer OS.	Kong et al., 2021
Germany	qRT-PCR	75	CRC patient tumor tissue	*F. nucleatum* abundance is not associated with overall OS (OR = 0.86, 95% CI 0.45–1.64, *P* = 0.86).	Galeano Niño et al., 2022
Ireland	RNA-seq	594	CRC patient tumor tissue	Elevated relative abundance of *F. nucleatum* is a favorable factor for disease-specific overall survival (OS) in mucinous CRC patients (HR 0.24, 95% CI 0.05–1.14, *P* < 0.05). The relative abundance of *F. nucleatum* has no significant impact on OS or disease-specific survival (DSS) in non-mucinous CRC patients.	Duggan et al., 2023
USA, Canada, Australia, New Zealand, Austria	qPCR	1,994	CRC patient tumor tissue	Patients with tumors containing *F. nucleatum* have a higher likelihood of dying from CRC compared to those without *F. nucleatum* (HR = 1.97, CI: 1.35–2.86, *P* < 0.05). The presence of *F. nucleatum* is not significantly associated with survival time (HR = 0.84, 95% CI: 0.21–3.34).	Borozan et al., 2022
Czech Republic	qPCR	129	CRC patient tumor tissue	Higher levels of *F. nucleatum* are associated with poorer OS compared to lower levels (adjusted HR 1.68, 95% CI 1.02–2.77, *P* < 0.05). The association between *F. nucleatum* and OS is significant in patients over 70 years old (HR 2.23, 95% CI 1.15–4.35, *P* < 0.05), in patients with left-sided tumors (HR 2.34, 95% CI 1.25–4.37, *P* < 0.05), and in patients who did not receive chemotherapy and/or radiotherapy (HR 1.87, 95% CI 1.02–3.45, *P* < 0.05).	Kunzmann et al., 2019
South Korea	qPCR	246	CRC patient tumor and adjacent non-tumor tissues	High *F. nucleatum* infection is associated with poorer overall survival in the palliative care group (26.4 vs. 30.7 months, *P* < 0.05).	Lee et al., 2018
Germany	qPCR	105	CRC patient tumor tissue	The abundance of *F. nucleatum* is not statistically significantly associated with OS.	Eisele et al., 2021
China	Droplet digital PCR	100	CRC patient tumor tissue	Patients with high *F. nucleatum* levels have significantly shorter overall survival compared to those with low *F. nucleatum* levels. Stage IV CRC patients with high *F. nucleatum* levels have significantly shorter overall survival compared to stage IV CRC patients with low *F. nucleatum* levels, with a sensitivity of 90.9% (95% CI 68.7–99.3%, *P* < 0.05) and a specificity of 88.9% (95% CI 66.4–98.6%, *P* < 0.05).	Yamaoka et al., 2018
China	qPCR	43	CRC patient tumor tissue	The recurrence rate is significantly higher in the *F. nucleatum*-positive group compared to the *F. nucleatum*-negative group (57.9% vs. 4.2%, *P* < 0.05). The abundance of *F. nucleatum* has a higher predictive value for CRC recurrence compared to the AJCC model (AUC = 0.75 vs. AUC = 0.738, *P* < 0.05).	Li et al., 2024
Spain	RNA-ISH	143	CRC patient tumor tissue	*F. nucleatum*-positive patients have a significantly higher risk of late recurrence after nCRT (HR = 7.1, 95% CI: 2.8–18.0, *P* < 0.001).	Serna et al., 2020
China	qPCR	30	CRC patient tumor tissue	Recurrence patients have enriched *F. nucleatum* in CRC tissues compared to non-recurrence patients (*P* < 0.05). The disease-free survival time differs significantly between low and high *F. nucleatum* groups (HR = 11.79, *P* < 0.05). The AUC of the *F. nucleatum* prediction model is higher than that of the AJCC staging (0.875 vs. 0.800, *P* = 0.001).	Wang et al., 2024
China	qPCR	92	CRC patient tumor tissue	High *F. nucleatum* levels are closely associated with shorter disease-free survival. The five-year recurrence-free survival rate is significantly shorter in the high *F. nucleatum* group compared to the low *F. nucleatum* group. The AUC for predicting potential CRC recurrence using *F. nucleatum* is higher than that of the AJCC staging model (0.776 vs. 0.646, *P* = 0.039).	Yu T. et al., 2017

## 6 Conclusion and prospects

The geographic variability in the association between *F. nucleatum* and overall survival (OS) warrants in-depth investigation. Chinese cohort studies demonstrate a significant association between high *F. nucleatum* abundance and poor overall survival (OS), whereas German cohorts show no such correlation. This heterogeneity may stem from three factors: (1) microbiota interaction differences due to cohort heterogeneity (*F. nucleatum* in Chinese populations may form synergistic pathogenic networks with enterotype microbiota through specific subspecies, while protective bacteria like Faecalibacterium prausnitzii in German cohorts may antagonize its pathogenic effects); (2) therapeutic strategy impacts (*F. nucleatum*-positive patients in Chinese cohorts receive adjuvant chemotherapy at lower rates, while German patients more commonly use targeted therapies that may obscure its prognostic value); (3) methodological differences in detection (Chinese studies predominantly use ddPCR for quantification, while German studies employ qRT-PCR, potentially affecting absolute quantification accuracy). These conflicting findings underscore the need for globally standardized *F. nucleatum* detection protocols and multi-center prospective studies to validate subspecies-specific prognostic value.

Based on multi-omics evidence, we propose the “*F. nucleatum* subspecies-specific pathogenic model” hypothesis: different *F. nucleatum* subspecies exert stage-specific regulation of CRC progression through differentially expressed core virulence factors (e.g., fnp gene clusters and fadA adhesins). Specifically: (1) *F. nucleatum* subspecies activates β-catenin signaling via the FadA/E-cadherin pathway, participating in CRC initiation and late-stage metastasis; (2) *F. animalis* subspecies primarily remodels the CRC immune microenvironment by modulating inflammatory and immune responses; (3) *F. vincentii* and *F. polymorphum* subspecies, currently detectable mainly in CRC tissues and saliva, lack mechanistic exploration in CRC pathogenesis. This model explains observed heterogeneities in subspecies distribution and prognosis, providing a theoretical basis for developing subspecies-specific diagnostic markers (e.g., 16S–23S ITS sequences for *F. animalis*) and targeted interventions (e.g., fnp gene cluster inhibitors).

We systematically integrated, for the first time, the multi-dimensional roles of *F. nucleatum* across the entire CRC diagnostic and therapeutic continuum. *F. nucleatum* influences CRC development not only by promoting tumor progression, lymph node metastasis, and distant metastasis but also innovatively establishes a complete clinical application framework from early screening to prognostic assessment: its non-invasive fecal detection potential offers a new strategy for early diagnosis, while quantitative PCR/immunohistochemistry-based methods have achieved precise correlation with AJCC staging. By integrating therapeutic interventions (e.g., antibiotics, phages) with efficacy monitoring indicators, we propose a closed-loop “detection-intervention-assessment” management model, offering a novel perspective for clinical translation research.

Future studies require deepening in three dimensions: (1) mechanistic dissection using multi-omics technologies to reveal interaction networks between *F. nucleatum*, the tumor microenvironment, and immune escape; (2) technical optimization through development of ultra-sensitive detection methods like CRISPR or digital PCR to enhance clinical applicability; (3) clinical validation via multi-center randomized controlled trials to confirm intervention efficacy (Wang et al., 2023). Furthermore, the “microbiota-host-therapy” trinity framework proposed herein will provide theoretical support for developing *F. nucleatum*-targeted personalized precision medicine strategies.
